# Determination of Variation Parameters as a Crucial Step in Designing TMT-Based Clinical Proteomics Experiments

**DOI:** 10.1371/journal.pone.0120115

**Published:** 2015-03-16

**Authors:** Evelyne Maes, Dirk Valkenborg, Geert Baggerman, Hanny Willems, Bart Landuyt, Liliane Schoofs, Inge Mertens

**Affiliations:** 1 Flemish Institute for Technological Research (VITO), Mol, Belgium; 2 CFP-CeProMa, University of Antwerp, Antwerp, Belgium; 3 KU Leuven, Research Group of Functional Genomics and Proteomics, Leuven, Belgium; 4 Interuniversity Institute for Biostatistics and Statistical Bioinformatics, Hasselt University, Diepenbeek, Belgium; Pacific Northwest National Laboratory, UNITED STATES

## Abstract

In quantitative shotgun proteomic analyses by liquid chromatography and mass spectrometry, a rigid study design is necessary in order to obtain statistically relevant results. Hypothesis testing, sample size calculation and power estimation are fundamental concepts that require consideration upon designing an experiment. For this reason, the reproducibility and variability of the proteomic platform needs to be assessed. In this study, we evaluate the technical (sample preparation), labeling (isobaric labels), and total (biological + technical + labeling + experimental) variability and reproducibility of a workflow that employs a shotgun LC-MS/MS approach in combination with TMT peptide labeling for the quantification of peripheral blood mononuclear cell (PBMC) proteome. We illustrate that the variability induced by TMT labeling is small when compared to the technical variation. The latter is also responsible for a substantial part of the total variation. Prior knowledge about the experimental variability allows for a correct design, a prerequisite for the detection of biologically significant disease-specific differential proteins in clinical proteomics experiments.

## Introduction

During the last decades, proteomics applications have been extensively used to elucidate biological/biomedical/clinical questions using differential protein expression approaches. Since the introduction of shotgun proteomics, a more high-throughput way of proteome analysis is possible compared to gel-based methods, making it feasible to analyze numerous samples. In these shotgun proteomics experiments, complex protein samples are enzymatically digested into peptides and the resulting mixtures are separated using liquid chromatography (LC), analyzed by tandem MS and submitted to database searching for identification [1]. Nowadays, the shotgun concept is expanded to a diversity of workflows not only at the level of separation and identification of proteins, but also in peptide/protein quantification methods [1,2]. However, in quantitative clinical proteomic studies, often little attention is paid to the determination of the workflow variability although these parameters are of utmost importance to determine an experimental design with sufficient statistical power to obtain confident disease-specific differential protein identifications [3].

To quantify proteins in clinical samples, isobaric tagging is one of the most popular labeling methods, as it allows multiplexing of up to ten different samples in one experiment [4]. Two kinds of amine-reactive isobaric tags are commercially available: tandem mass tags (TMT) (2-plex, 6-plex or 10-plex) and isobaric tags for relative and absolute quantification (iTRAQ) (4-plex or 8-plex). In these isobaric tags, N-hydroxy- succinimide chemistry is used to target free N-terminal and epsilon amino groups of lysine residues of peptides. Both TMT and iTRAQ reagents contain a reporter group and an amino-reactive group, spaced by a balancer group which generates an identical mass for all tags, hence isobaric. Relative quantification of the differentially labeled peptides is achieved by the generation of a reporter ion with a unique mass upon fragmentation of the peptide precursor. The ratio between the signal intensities of these reporter ions in the tandem mass spectra reflects the relative expression differences of the peptides in the different multiplexed samples [5].

Although multiplexing of several samples facilitates a direct comparison of the reporter ions, many aspects in this multistep workflow can be a source of variability, and the final output depends on a wide variety of factors. To use peptide-based TMT-labeling technology successfully in clinical proteomics, characterization of these sources of variation and the procedure-specific limitations is necessary. With this information, a framework for sample size determination can be proposed, resulting in a well-powered experimental design.

The most acknowledged limitations of isobaric labels are related to precision and accuracy. Recently, several research groups demonstrated that a lack of precision and accuracy of the reporter ion measurement in complex samples, due to co-selection of contaminants along with the precursor peptide in tandem mass spectrometry, complicates interpretation of quantitative data [6,7]. In these studies, proteins with known concentrations were spiked into the samples before labeling and both the precision and accuracy of these quantified ratios were investigated. In general, an underestimation of the actual ratios was observed due to this interference problem. Indeed, this contamination from the background results in an attenuation of the expression differences in the sample and perturbs the actual abundance ratios. However, the isobaric labeling strategy is still useful to detect true differences in protein expression. Since the background contaminants force the peptide ratios closer to unity, an assessment of variability is even more important to design an optimal experiment with sufficient statistical power to detect the expression difference.

In this pilot study, we try to find the most optimal conditions to allow large-scale quantitative clinical proteomics experiments. In our approach, we determine several variation parameters (total, technical and labeling variation) linked to the use of a TMT-sixplex proteomic platform in a limited number of real clinical samples. For this purpose, we used human peripheral blood mononuclear cells (PBMC), a complex cellular sub-fraction isolated from whole blood. Because PBMCs are the main actors in several inflammatory processes, they can be linked to several diseases [8,9]. Global proteomic profiling of these cells is therefore of high interest. Although several proteome studies already have commented on this cell type [10–13], none of these studies used gel-free isobaric TMT labeling.

## Materials and Methods

### Ethics statement

The blood samples were taken with the approval of the local ethical committee (Ethical committee UZA (Antwerp University Hospital), No. 12/7/69) and a signed informed consent from every volunteer is available.

### PBMC sampling

Blood from 6 healthy volunteers (3 females, 3 males, ages 50–60, with no clinical/laboratory signs of inflammation) was collected in 2x 10 ml k_2_EDTA vacutainers (BD, Erembodegem, Belgium). PBMCs were isolated from these blood samples within 2 hours after blood withdrawal. To isolate PBMC cells, leucosep tubes (Greiner Bio-One, Wemmel, Belgium) were used. Blood was diluted 1:2 with Dulbecco’s Phosphate Buffered Saline (PBS) (Sigma, St Louis, Missouri) prior to transferring it into the leucosep tube. After centrifugation (10 min, 1000 *g* and ambient temperature), the PBMC cell layer of two leucosep tubes were pooled and transferred into a 15 ml falcon tube. To wash the PBMCs, the sample was diluted with 10 ml PBS and centrifuged for 10 min at 250 *g* and ambient temperature. This step was repeated twice. The obtained cell pellets were stored at −80°C until further use.

### Sample preparation

The PBMC cell pellet was lysed using RIPA buffer (1x) (Thermo Scientific, Rockford, IL) containing also 1x HALT phosphatase inhibitor (Thermo Scientific) and 1x HALT protease inhibitor (Thermo Scientific), combined with a 30 s during sonication (Branson Sonifier SLPe ultrasonic homogenizer, Labequip, Ontario, Canada) of the sample on ice. After centrifugation of the samples for 15 min at 14,000 *g* on 4°C, the pellet was discarded. To improve further solubilisation of the proteins and to provide an efficient digestion, 0.1% Rapigest SF surfactant (Waters, Milford, MA) was added to the supernatant and the sample was incubated for 5 min at 100°C. Next, the protein concentration was determined using the Pierce BCA protein Assay kit (Thermo Scientific).

Before labeling the samples, 60 μg protein of each sample were reduced using 1.25 μl of 500 mM tris(2-carboxyethyl) phosphine, supplied with the TMT labeling kit (Thermo Scientific), in a volume of 100 μl 100 mM TEAB, and incubated for 1 h at 55°C. Next, the samples were processed using the filter-aided sample preparation (FASP) procedure (FASP Protein digestion kit, Protein Discovery, Knoxville, TN) according to manufacturer’s instructions. In short, the samples were diluted in a urea buffer and processed on a FASP filter, alkylated with iodoacetamide and digested with trypsine (enzyme:protein ratio = 1:50) overnight. Afterwards, the tryptic digests are desalted using Pierce C18 spin columns (Thermo Scientific) according to manufacturer’s instructions. Next, the eluted peptides were vacuum dried and reconstituted in 100 mM TEAB to a final concentration of 1μg μl^−1^ before labeling was performed.

### TMT labeling

For the reconstitution of the tags, the TMT labels were dissolved in 41 μl acetonitrile according to the manufacturer’s protocol. From every sample, 10 μg was labeled with 4.1 μl of a TMT tag dissolved in acetonitrile and every sample was incubated for 1 hour at ambient temperature. The labeling reaction was stopped by adding 2 μl 5% hydroxylamine. After 15 minutes, a pooled sample was prepared based on the six labeled samples with a protein concentration ratio of 1:1:1:1:1:1. To remove the excess of labels, the pooled sample was cleaned again using C18 spin columns and vacuum dried. The experimental design of the TMT labels is discussed in the section on “Set-up of the study”.

### Nano reversed phase liquid chromatography and mass spectrometry

The peptide mixture was separated by reversed phase chromatography on an Eksigent nano-UPLC system using an Acclaim C18 PepMap100 nano-Trap column (200 μm x 2 cm) connected to an Acclaim C18 analytical column (75 μm x 15 cm, 3 μm particle size) (Thermo Scientific, San Jose, CA). Before loading, the sample was dissolved in mobile phase A, containing 2% acetonitrile and 0.1% formic acid, and spiked with 20 fmol Glu-1-fibrinopeptide B (Glu-fib, Protea biosciences, Morgantown, WV). A linear gradient of mobile phase B (0.1% formic acid in 98% acetonitrile) from 2 to 35% in 50 min followed by a steep increase to 98% mobile phase B in 2 min was used at a flow rate of 350 nl min^−1^. The nano-LC was coupled online with the mass spectrometer using an PicoTip Emitter (New objective, Woburn, MA) coupled to a nanospray ion source (Thermo Scientific).

The LTQ Orbitrap Velos (Thermo Scientific, San Jose, CA) was set up in a MS/MS mode where a full scan spectrum (400–2000 m/z, resolution 60,000) was followed by a maximum of five dual collision-induced dissociation (CID)/ high-energy collision-induced dissociation (HCD) tandem mass spectra (100 to 2000 m/z) [14,15]. Peptide ions were selected for further interrogation by tandem MS as the five most intense peaks of a full-scan mass spectrum. CID scans were acquired in the linear ion trap of the mass spectrometer, HCD scans in the orbitrap, at a resolution of 7500. The normalized collision energy used was 35% in CID and 55% in HCD. We applied a dynamic exclusion list of 90 s for data dependent acquisition.

### Data analysis

Proteome discoverer (1.3) software (Thermo Scientific, San Jose, CA) was used to perform database searching against the international protein index (IPI) Human 3.87 database using both the Sequest and Mascot algorithms. Following settings were applied: precursor mass tolerance of 10 ppm, fragment mass tolerance of 0.8 Da. Trypsin was specified as digesting enzyme and 2 missed cleavages are allowed. Cysteine carbamidomethylation and TMT modifications (N-terminus and lysine residues) were defined as fixed modifications and methionine oxidation and phosphorylation (STY) were variable modifications. The results were filtered using following settings: only medium and high confident peptides with a global FDR < 5% based on a target-decoy approach [16] and first ranked peptides were included in the results. In the TMT quantitation workflow the most confident centroid method was used with an integration window of 20 ppm. The extracted reporter ion intensities of all the quantification channels in all the runs were transformed by the logarithmic function with base 2. The log_2_-transformed intensity distribution was normalized by shifting the centre (median) of the distribution to the median of the median log_2_-intensity values in all the quantification channels over all the LC-MS runs. The median was chosen as a robust statistic against outliers.

### Set-up of the study

**Fig 1:**
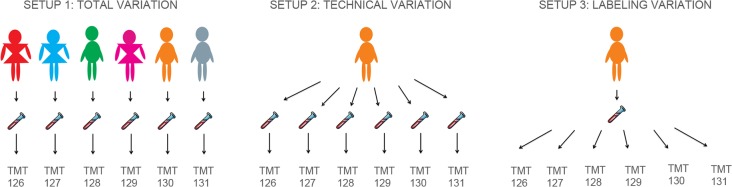
Overview of the experimental designs. Visualization of the three different set-ups in our variation study.

The PBMC samples of 6 healthy volunteers (3 females, 3 males, and ages 50–60, with no laboratory /clinical signs of inflammation) were used to conduct LC-MS experiments with different strategies for the TMT labeling. The experimental design is displayed in Fig. 1. First, to evaluate the total variation, that includes the biological variability, (Fig. 1A), we applied TMT labeling to the PBMC fractions of 6 healthy volunteers. After blood withdrawal and subsequent processing of the PBMCs of 6 different persons, the six samples were labeled with 6 TMT tags with different reporters (called TMT 126 to TMT 131). It should be noted that this variation also includes the variation due to sample preparation and labeling. Second, to establish technical variation settings (Fig. 1B), six distinct PBMC isolation procedures from a blood withdrawal of one of the previously committed healthy volunteers were performed. Each sample was processed separately and labeled prior to pooling and analysis on LC-MS. This experiment captures the technical variation due to sample preparation and labeling. Third (Fig. 1C), one of the previously processed PBMC samples was subdivided in 6 parts and labeled with the six different TMT tags prior to pooling. We argue that this experiment truly captures the labeling variation and reporter ion generation as the labeling chemistry is equal for the six labels. In this way, an estimation can be made regarding the labeling variation.

## Results

In this pilot study, a clinical proteomics platform was optimized for PBMC specimens and the different sources of variation involved in this gel-free isobaric labeling proteomic procedure were characterized. Through this evaluation, we can ensure that experimental design and data analysis are optimal for reproducible large-scale biomarker discovery studies. In order to obtain results of high quality, Glu-1-fibrinopeptide-B (Glufib) was used as a standard peptide for quality control of the LC-MS system. In our experiment, this peptide was added after the TMT labeling was performed (prior to injection of the sample in the LC system), and thus reflects the performance of the LC-MS/MS part of the experiment.

As quality control of LC-MS runs cannot estimate the variance within a TMT experiment, a series of ‘variation’ experiments using TMT reagents were set up. While multiplexing ensures that pooled samples are affected by the same amount of noise such that a direct statistical analysis is possible, other variations within the lay-out of the experimental design are still interfering with the signal intensities. To evaluate the extent of total, technical and labeling variations, we proposed three different experimental set-ups according to Fig. 1.

To allow a high-precision measurement of the TMT reporters, we opt to use the parallel CID/HCD approach, developed by Kocher *et al*.[15]. In this procedure, the MS^1^ survey scan is acquired in the orbitrap analyser, followed by a concomitant fragmentation by CID and HCD, with fragment ion analysis in the LTQ and orbitrap part of the mass spectrometer, respectively. By using this set-up, a high precision of reporter ion quantification is ensured, while maintaining qualitative fragment spectra for peptide identification (CID).

To achieve a relevant number of identified and quantified peptides, the samples were separated on a nano-LC (reversed phase) gradient of 3 hours and a data-dependent acquisition method which selected the 5 most intense peaks for subsequent parallel CID/HCD analysis, was applied. Note that each sample was measured in triplicate to establish the reproducibility of these measurements. The raw data were interpreted with both Sequest and Mascot algorithms and the identified and quantified peptides were carefully filtered. Only medium or high confident peptides were used and only first ranked peptides were allowed. Furthermore, to avoid problems with peptides that are shared by multiple proteins, only unique peptides were taken into account. Finally, all peptides with no quantification values or with the absence of one or more reporter ions were also removed. The distributions of the missing reporters per sample are displayed in S1 Table.

**Fig 2:**
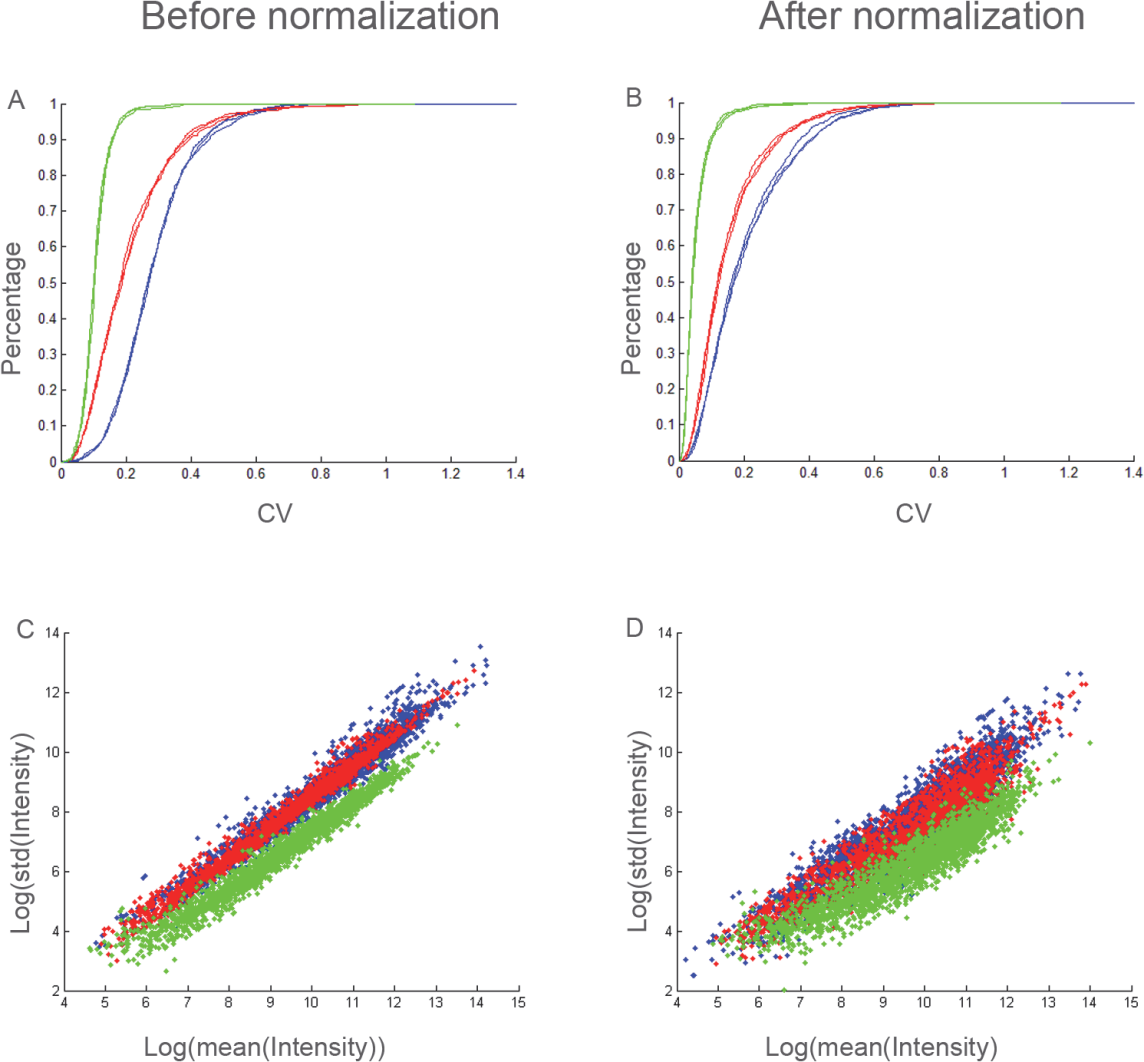
Comparison of coefficient of variations in the three different set-up: labeling (green), technical (red) and total (blue) variation set-up. Upper panels (A+B): Plot of coefficient of variation versus the percentage of peptides. The peptides included in these graphs are those present in the intersection of the triplicate runs. The curves after normalization (B) are shifted more to the left, indicating a reduction of the variability. Lower panels (C+D): Comparison of log_2_(standard deviation) of these ‘intersection’ peptides as a function of the log_2_ (mean reporter intensity). The scatterplot demonstrates that removing the systematic effects that drive the CV by normalization, results in more variability between the peptides.

Next, the coefficient of variation (CV) of the identified and quantified peptides in the different proposed set-ups was calculated, i.e. for each identified peptide the standard deviation of the six corresponding reporter ion intensities were divided by the mean of the six reporter intensities. Because a shotgun proteomics experiment cannot regulate that the same set of peptides are identified across different LC-MS runs, the intersection of confident identifications of the three triplicate runs was taken as a ‘core peptide pool’ for each experimental set-up, i.e. total, technical and labeling. Although both search engines, Sequest and Mascot, were used for identification purposes, further calculations were performed only using the Mascot peptide identification set. However, the same observations are seen using the Sequest dataset (data not shown). As a result 1028 non-redundant peptides were preserved for the three replicate runs of the total variation set-up, 854 peptides for the technical variation and 848 peptides were found in the intersection of the triplicate runs of the labeling variation set-up. A visualization of the CV of these peptides in function of the cumulative percentage of the peptides having a maximum CV, can be found in Fig. 2A. Here, a clear distinction within the different set-ups is visible. The graph illustrates that the labeling variation (green) is significantly lower compared to both technical (red) and total variation (blue) set-ups. Also, the calculated CV distributions of triplicate injections of each set-up are very comparable, indicating that reporter ion generation within a sixplex experiment is a reproducible procedure in a TMT-oriented parallel CID/HCD LTQ-Orbitrap Velos workflow.

However, no normalization was implemented here yet. To establish whether normalization was needed, the intensity values of the six reporters of all quantified peptides in the triplicate runs of the three different set-ups were visualized using boxplots (S1 Fig.). Although in general no abnormal fluctuations of the boxplot medians were observed, normalizing the data through ‘median-normalization’ is able to remove the systematic effect (e.g. pipetting errors) in a very convenient way (S1 Fig.). More advanced normalization schemes, such as quantile normalization or intensity-based normalisation are possible as well. A review on available normalization techniques suited for LC-MS is provided by Ejigu *et al*. [17].

Using these normalized data, the CV values of the peptides were recalculated, using the same settings for the three different set-ups as before normalization. In this way, the influence of normalization on the variance values in the proteomics set-ups can be established. Fig. 2B represents the normalized CV values as a function of the percentage of peptides that have a variability below the corresponding CV value. Differences between these two figures (before and after normalization) are noticeable as the curves after normalization are shifted more to the left, indicating a reduction of the variability. This reduction in the overall variation illustrates that systematic effects, such as small pipetting errors during labeling, can have major influences in the overall variation. Next, also the standard deviation, in fact log_2_(standard deviation), of these peptides in function of the log_2_(mean reporter intensity) is visualized before and after normalization for one arbitrary set of replicates (Fig. 2C and 2D) for total (blue), technical (red) and labeling (green) set-up. Again, normalization alters the distribution of the data points. From the plot two conclusions can be drawn: first, the standard deviation increases with the intensity, indicating a heteroscedastic error structure. Second, the standard deviation decreases after normalization, but the plot suggests that the variability between the peptides, i.e., points in the plot, is inflated. This observation is not unexpected as biological variability is no longer obscured by systematic effects in the data.

**Fig 3:**
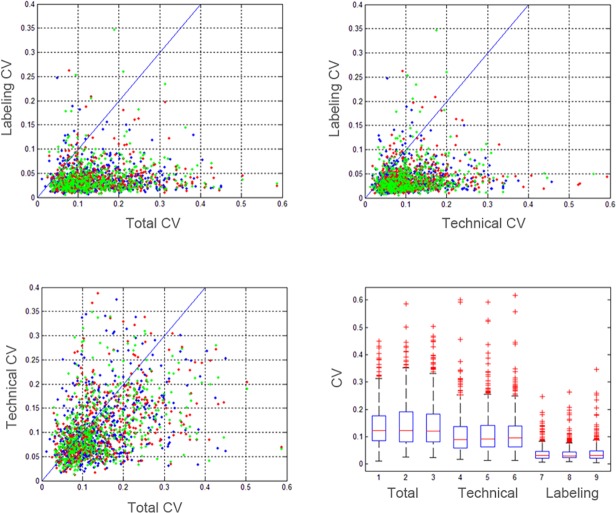
Comparison between total, technical and labeling CV of ‘core’ PBMC peptides. The peptides of the three replicates are represented in three different colors (red, green, blue). Most peptides have a lower labeling CV compared to both technical and total CVs. The comparison of technical CVs versus total CVs does indicate that some peptides show higher technical CVs compared to total CVs, indicating that technical issues contribute a substantial part to the total variation. The boxplot representation clearly demonstrates that higher median peptide CVs are present in the total CVs compared to technical CVs and labeling CVs.

To further elucidate this observation, we searched for those confidently identified and quantified peptides which were present in all triplicate injections of the 3 set-ups. As shown by Fig. 3, 549 peptides were in common between the total, technical and labeling set-up for all replicates. These peptides represent probably quite abundant peptides, as they are reproducibly identified in data-dependent acquisition mode. Within these 549 peptides, the correlations between total, technical and labeling CV are visualized pairwise in Fig. 3 for the three replicates, indicated by a color code. As expected, the majority of the 549 peptides shows a substantially lower labeling CV in comparison to both total and technical variation values. Comparison of total versus technical CV, on the other hand, reveals that some peptides have high CV values in the total variation set-up and low CV values in the technical set-up and others have high technical CV values but lower total CVs. Although more peptides have a higher total variation than technical CV, these data illustrate that the variation due to sample preparation contributes for a substantial part to the overall variation for the set of peptides identified in each of the three sets of experiments. In Fig. 3D, an alternative visualization with boxplot representation demonstrates the differences of these normalized peptide CV values in the different set-ups.

**Fig 4:**
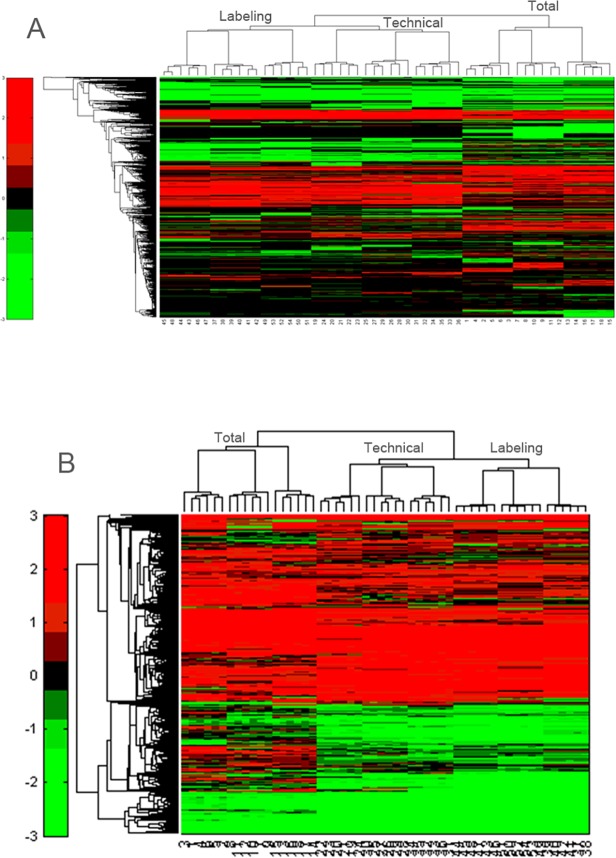
Clustering analysis. Hierarchical clustering on the ‘core’ dataset (A) as well as on the union (B) of all identified and quantified peptides.

Next, hierarchical clustering was performed both on the ‘core’ dataset (intersection) (Fig. 4A) as well as on the union (Fig. 4B) of all identified and quantified peptides. In this figure, the color code represents the log_2_ intensities of all peptides minus the global median (= 14.1089)). Here, every sample is numbered. The total variation set-up starts from number 1 (= first sample in sixplex in first replicate run labeled with TMT126) until number 18 (= sixth sample in sixplex in third replicate run labeled with TMT131). The technical (No. 19–36) and labeling set-up (No. 37–54) are numbered in the same way. The hierarchical clusters demonstrate that the peptides are nicely clustered per run and per set-up. Moreover, the length of the branches in the dendrogram also illustrates that, the technical and labeling set-ups are more tightly grouped than the total set-up. This observation reflects the set-up of the experiment, as both the technical and labeling set-up are based on sample material from the same individual.

**Fig 5:**
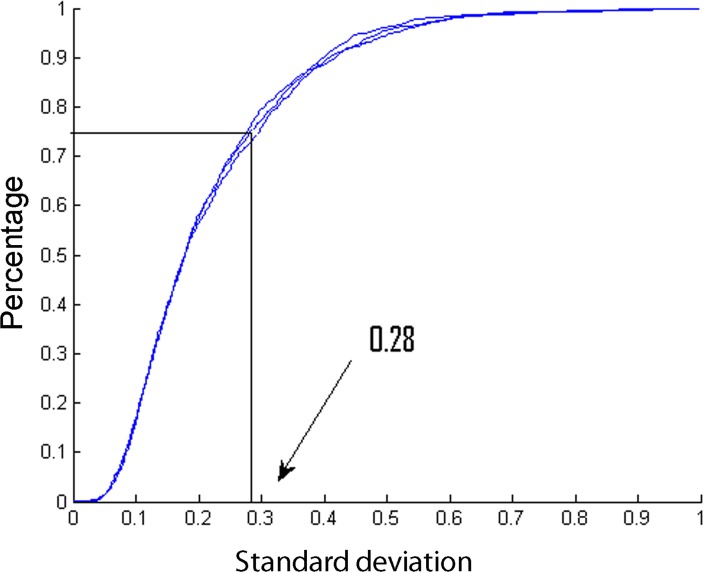
Representation of the percentage of peptides of the total variation set-up in function of the standard deviation of log intensity values. The sample was injected in triplicate, indicated by color code blue. This graph demonstrates that 75% of the peptides in the three replicate runs do not have a standard deviation that exceeds 0.28. These numbers can be used in sample size calculations.

Information about the variability was further used to calculate the sample size needed to obtain statistical relevant results using this platform. Fig. 5 shows a graph representing the percentage of peptides (Y-axis) of the total variation set-up in function of the standard deviation of log intensity values (x-axis). This graph demonstrates that 75% of the peptides in the three replicate runs do not have a standard deviation that exceeds 0.28. With this information, a sample size calculation can be determined as described in [18]. The sample size required to detect a peptide fold change of two between PBMC fraction of healthy volunteers and patients in the 75% least variable peptides with a two-sided 0.001 significance level with 90% power is equivalent to 7 subjects in each group. It should be noted that in this calculation we assume that the variability in the healthy group is representative for the variability in the patient group. However, since the variability might slightly change in function of patient’s condition, additional persons could be recruited to remediate uncertainty about patient variability.

## Discussion

This study evaluates the potential and technical merits of TMT-based quantitative proteomics of PBMCs. In this quantitative proteomics workflow, the importance of determining the sources of variation is sometimes minimized, however, important inter-individual differences in protein expression are seen in the human population [19]. These variations have major implications for the experimental design of a study, as variations influences statistical power [3,10]. To avoid false discoveries driven by ‘underpowered’ quantitative proteomics experiments, it is essential to determine the global variation in real clinical samples prepared by the preferred method. Evidently, it is extremely important that the detected changes in clinical proteome studies are true biological differences as further validation of these targets with complementary techniques is costly and time-consuming. Therefore, the need to develop a reliable workflow for clinical proteomics procedures and to determine the sources of variation in the workflow is evident. In this work, we describe an analytical set-up that can be applied for the identification of variability arising from total, technical and labeling issues across the quantitative TMT workflow.

The use of gel-free isobaric tag proteomic procedures is, in general, an attractive method for clinical biomarker discovery. However, in spite of its many advantages, quantitation with isobaric tags is not without limitations. Recently, some issues about precision and accuracy of isobaric labels have been reported [6,7]. A paper by Ow *et al*. showed how iTRAQ suffers to some extent from the compression of the quantitation ratios to a unity ratio as a result of co-selection of background peptides during precursor ion selection in complex samples [20]. Karp and colleagues also described accuracy and precision issues in iTRAQ quantification and proposed a mathematical solution to overcome the underestimation of the protein ratios [7]. Recently, Ting *et al*. proposed a method which results in an almost complete elimination of this ratio distortion [21]. By implementing an additional round of fragmentation (resulting in MS^3^ spectra), a significant reduction of interference with ion selection is obtained [21]. This approach however, has two main drawbacks: 1) the method depends on Lys-C for protein digestion, resulting in longer proteolytic peptides and 2) the additional fragmentation round requires increased mass spectrometer cycling time, resulting in a reduced number of protein identifications [6].

We argue that the distortion of the peptide ratio to unity due to co-isolation of background contaminants is indeed a drawback, but that this effect does not undermine a differential analysis of protein expression. A valid proteome analysis is therefore still possible when an adequate number of individuals are recruited to ensure sufficient power to detect these diminishing differences between the different experimental groups. To address these accuracy and precision issues in our set-up, we applied the strategy of Kocher *et al*. [15] in our LTQ orbitrap Velos system, where low and high energy tandem mass spectra are combined. Due to the decoupling of the identification (via CID in LTQ part of the instrument) and quantification (via HCD in orbitrap analyzer of the instrument), different fragmentation energies and high resolution measurements of reporter ions are possible, resulting in high precision of TMT reporter tags. Because, in our hands, the MS^3^ method resulted in a significantly reduced number of protein identifications, we opt to still perform MS^2^ measurements for discovery studies, but take into account that an under-estimation of the ratios is possible. This approach might be debatable, but with this method, the general trends of up- or down-regulation are still correct.

To examine the ‘optimal’ conditions for PBMC proteomics, the different sources of variation involved in gel-free isobaric labeling proteomic procedures were determined. The design of the study allows the investigation of total, technical and labeling variation of PBMC proteomics workflow (Fig. 1). Comparing these three measures gives information crucial for obtaining statistically relevant information for future quantitative proteomic measurements. Because a data-dependent acquisition method was used, several peptides were identified in only one of the triplicate runs. With the bulk of the peptides (95%) below an overall CV of 0.5 (Fig. 2), highly variable peptides between the 6 different individuals were only present in limited numbers.

Nonetheless, performing normalization has, as shown in Fig. 2, consequences in variation values of peptides. The trend in Fig. 2C and 2D for example, demonstrates that removing the systematic effects that drive the CVs results in more variability between the peptides. Although this suggests that between-peptide variability is less homogeneous after normalization. This finding does not impose a problem for biological samples, as the overall variation decreases. For example, low labeling variation is present before (Fig. 2A) and after (Fig. 2B) normalization. However, in the normalized dataset more than 90% of the peptides have a CV below 0.1, instead of only 40% in the dataset that is not normalized. This trend indicates that small pipetting errors or errors in the determination of protein concentration can have major consequences, when not taken into account. These data also illustrate that introducing a chemical labeling step into the proteomics workflow, has an almost negligible effect on the obtained overall variation, when correct normalization procedures are applied.

The contribution of technical sample preparation steps to the total variation is far more pronounced. Figs. 2 and 3 clearly show that only a small difference is seen between isolation of PBMCs from 6 different individuals (which can be used to determine interindividual variation) and the technical variation, calculated from 6 independent PBMC isolations from one healthy person. However, in one of our previous studies we could already conclude that isolation of PBMCs from whole blood and additional sample preparation is a major factor in causing variation between samples [10]. Although the noise contribution from the sample preparation is substantial, hierarchical clustering shows that the grouping is still driven by biology as on the peptide level, substantial differences between the different set-ups can be found. However, we should mention that the clustering of the union is probably driven by the missingness pattern, which also results in clustering per run.

In recent years, the importance of establishing factors of variations in different workflows has become clear. Several research groups tried to elucidate these variation questions by means of gel-based proteomics [10,22–24]. In gel-free approaches, the importance of identifying the sources of variation became obvious only recently. Initially, studies using isobaric tags tried to understand the potential sources of variation, as adding labels might also increase the overall variability. In 2005, Gan and co-workers assessed the reliability of iTRAQ through monitoring of technical, experimental and biological variations using three bacterial model systems. In their strategy, CV-values based on protein levels were determined (in contrast to our study were peptide information was used). They concluded that the average technical variation (CV = 11%) was lower compared to the average biological variation (CV = 25%) and that the contribution of the MS variance was negligible [25]. In 2008, Song and colleagues provided an experimental design using iTRAQ in plasma biomarker discovery. Here, standard deviations and CV were calculated based on protein ratios. In this study, the variance between 4 healthy volunteers compared to a pooled standard was established. The authors found that 90% of the quantified proteins did show a standard deviation below 0.6 in their ‘biological’ variation set-up and a standard deviation of 0.3 was attributed to ‘technical’ issues [26]. Although the study was applied on real clinical samples, we do call biological variation, total variation, because not only differences in biological parameters (e.g., gender, age, health status,..) but also all technical issues are included in this variation factor. More recently, Zhou and colleagues also investigated the temporal, technical and biological variability in quantitative plasma proteomics studies using iTRAQ. Even though their overall variation values (CV>60%) were quite high, working with the 70% least variant proteins seems manageable for plasma biomarker studies [27]. However, besides plasma, no other studies with clinical samples were conducted to establish variation in a workflow with isobaric labels. In our approach, we perform the assessment of variation in PBMCs on the level of peptides to obtain an unbiased view on the sources of variation, since any effects observed on this layer will also influence subsequent quantification of proteins. However, different protein inference strategies can interfere with the quantification step, which makes a comparison less transparent.

In label-free quantitation approaches, the variation in proteins/peptides extracted from several other clinical sample types was determined. Because of the absence of labels in these workflows, the instrumental variability becomes more important in these studies. Nagaraj *et al*. compared the intra- and interindividual variability of the urinary proteome using a label-free platform. Although their instrumental CV had a median value of 18%, the intra- and interindividual variation reached much higher median values of 48% and 66% respectively [28]. Piehowski *et al*. evaluated 4 critical steps that contribute to the overall variability in the analysis of proteomic human brain tissue samples: extraction, digestion, instrumental stability and instrumental variability. Based on peptide quantification, they found an overall median CV of 34%. Besides the extraction with the highest variability, a substantial part of the variance is due to the instrumental stability (median CV of 17%) [29]. Also Perrin and colleagues applied label-free proteomics and studied the technical and inter-individual variation of cerebrospinal fluid samples [30]. Using pooled samples, and thus reducing biological variation substantially, they could achieve very low CV values for 81 proteins (CV <5%). Other approaches, including the establishment of the technical variation factors in metabolic labeled shotgun approaches [31] and theoretical models for statistical design of quantitative proteomics studies [32] have also been published.

Our study demonstrates that knowledge about interindividual variation and technical issues can help to design a proteomics experiment with sufficient statistical power. We could show that, at least 7 biological independent PBMC samples per experimental group are needed to find 2-fold changes in peptides with a two-sided 0.001 significance level and with a statistical power of 90%. The ability to perform sample size calculations using several experimentally determined parameters, including standard deviations, may help to find disease-specific marker candidates in future proteomic (biomarker discovery) studies.

Indeed, this non-targeted discovery phase is only the first step in the biomarker pipeline, and a costly verification and validation step is required before a biomarker panel can be implemented in a clinical setting [33]. Obtaining biomarker candidates from a well-designed discovery study is therefore of utmost importance, as the development of alternative methods (e.g. multiple reaction monitoring (MRM) measurements or antibody-based validation procedures (e.g. enzyme-linked immunosorbent assays (ELISA) or Western blot) takes a lot of time and money [34,35], and it thus only worth it with reliable candidates rather than false-positive marker candidates.

In conclusion, we can say that performing quantitative experiments with complex samples remains a challenge, but that it remains a valid approach in biomarker research if a number of factors can be kept under control. To minimize the false positive identification rate in shotgun TMT-based quantitative proteomics, an unbiased design and adequate statistical power is needed. This statistical power is influenced by the variability, sample size and the requested significance level and fold change. Understanding the overall variation of the proteomics method used, makes it possible to determine the sample size needed. With careful experimental design, the strengths of isobaric labeling outweigh their weaknesses. Including these parameters in future PBMC biomarker studies, increases the chance of finding real disease-related differences, and thus potential markers which might meet the high bar of clinical validation.

## Supporting Information

S1 Fig Boxplot representation of the intensity values of the six reporters of all quantified peptides in the triplicate runs and three different set-ups. The reporter channels are numbered starting from 1 (= TMT126 of first set-up (total) and first replicate) towards 54 (= TMT 131 of third set-up (labeling) and third replicate).
(TIF)

S1 Table Missing quantification data. Overview of missing reporters (quantification channels) per experiment for all identified peptides (FDR 5%) using MASCOT.
(XLSX)
