# Supplementary figures and images for: Determination of Variation Parameters as a Crucial Step in Designing TMT-Based Clinical Proteomics Experiments
Source: PLoS One. 2015 Mar 16;10(3):e0120115. doi: 10.1371/journal.pone.0120115 (PMC4361338; doi:10.1371/journal.pone.0120115)

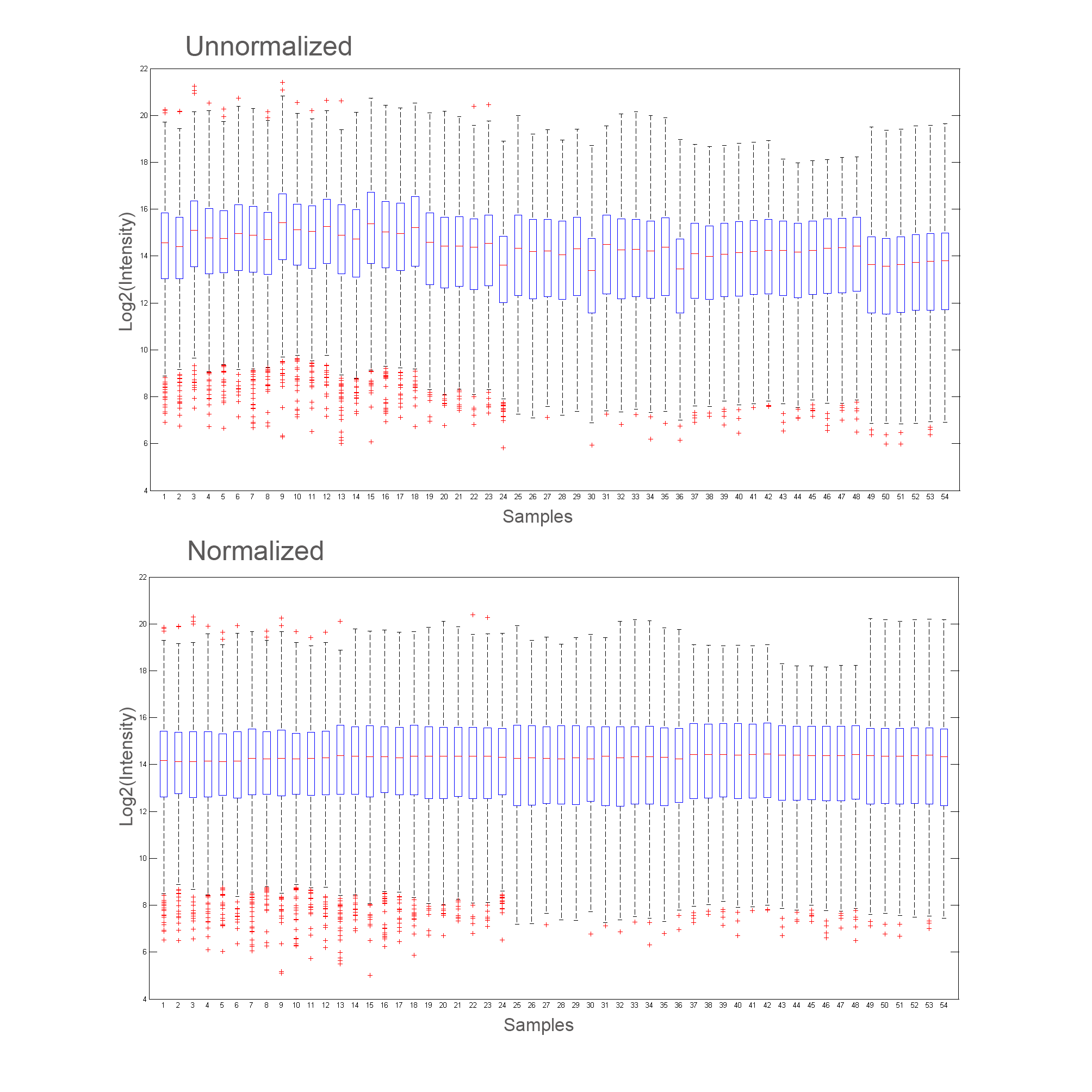

Supplement: S1 Fig — The reporter channels are numbered starting from 1 (= TMT126 of first set-up (total) and first replicate) towards 54 (= TMT 131 of third set-up (labeling) and third replicate). (TIF) [file pone.0120115.s001.tif]
